# Rural-Urban Variation in Weight Loss Recommendations Among US Older Adults with Arthritis and Obesity

**DOI:** 10.3390/ijerph16060946

**Published:** 2019-03-16

**Authors:** Mary L. Greaney, Steven A. Cohen, Christie L. Ward-Ritacco, Deborah Riebe

**Affiliations:** 1 Health Studies, University of Rhode Island, 25 West Independence Way, Kingston, RI 02881, USA; steven_cohen@uri.edu; 2 Department of Kinesiology, University of Rhode Island, 25 West Independence Way, Kingston, RI 02881, USA; christieward@uri.edu (C.L.W.-R.); debriebe@uri.edu (D.R.)

**Keywords:** arthritis, older adults, obesity, rural-urban, weight loss recommendation

## Abstract

*Purpose*: Weight loss is advantageous for individuals with obesity and arthritis. Therefore, this study was conducted to determine if there are differences by rural-urban status among older adults with these conditions who reported being advised by a health care provider to lose weight for arthritis or to ameliorate arthritis symptoms. *Methods*: A cross-sectional analysis of 2011 Behavioral Risk Factor Surveillance System (BRFSS) data. Respondents reported if they had been diagnosed with arthritis and if they received a provider weight loss recommendation (WLR). The analytic sample was limited to older adults aged 60–79 living in the five states that administered the examined BRFSS arthritis module who had body mass index ≥ 30 kg/m^2^ and reported having arthritis (n = 2920). The respondent’s county of residence was linked to the corresponding county-level population density from the US Decennial Census to determine rural-urban status. A generalized linear model examined the association between receipt of a WLR and population density, controlling for demographics. *Results*: The sample was 83.6% white, 57.8% female, and 63.2% received a WLR. Respondents from more urban counties were more likely to receive a WLR (*p* value for trend <0.001). Additionally, older respondents, men, individuals with less than a high school education, and whites had a decreased likelihood of receiving a WLR. *Conclusions*: The analysis identified notable rural-urban differences with respondents in more urban counties being more likely to receive a WLR. Furthermore, there were differences in those who received a WLR by age, sex, and education. Reasons for these differences should be explored.

## 1. Introduction

The United States (US) population is aging and it is estimated that one in five US adults will be 65 or older by 2030. An estimated 41.0% of US adults aged 60+ are overweight or have obesity [1], making prevention and reducing obesity a critical public health issue. Obesity is an underlying cause of numerous health issues across the lifespan [2], including arthritis [3,4,5,6]. Due to the increasing aging population and rising obesity rates, it is projected that 78.4 million US adults will have arthritis by 2040 [7], and this will likely decrease quality of life, increase rates of disability and the use of nursing home care, and have significant financial impacts on medical expenditures [5,8].

Arthritis is the most prevalent medical condition in individuals over 65 years old, with approximately 50% of this age group being affected [9,10]. Furthermore, obesity has increased among older adults with arthritis. Barbour and colleagues’ analysis of the 2009–2014 National Health Interview Survey data determined that the unadjusted obesity prevalence increased among older adults with doctor-diagnosed arthritis from 29.4% in 2009 to 34.3% in 2014 [11]. Reducing obesity among individuals with arthritis is important as weight loss can ameliorate arthritis symptoms, reduce pain, improve physical function, and increase quality of life [5,12,13].

Physician counseling is associated with increased intention to lose weight and weight loss [14,15]. Nonetheless, research indicates that more than half of patients who are overweight or have obesity in the US have not received physician counseling for weight loss [15,16,17,18]. A recent study of primary care patients with overweight/obesity found that 59% had been advised by their physician to lose weight [19]. While this is encouraging, it also indicates the need for increased weight loss counseling by healthcare providers. Analysis of data from the National Physical Activity Weight Loss Survey found that physician counseling for weight loss decreases with increasing age among older adults; 41.8% of respondents aged 60–69 and 30.5% of those aged 70–79 with obesity were counseled to lose weight [18]. Previous studies have found that individuals with overweight or obesity are more likely to receive physician counseling for weight loss if they have a higher income [20,21], greater educational attainment [21], have obesity (versus overweight) [18,22], are female [18], have a chronic disease(s) [18], live in the Northeast and South (vs. the West), and are older (aged 40–49 vs. 18–29) [18]. Recent analysis determined that from 2001 to 2014 there was a 10.4 percentage point increase (from 35.1% to 45.5%) among adults 18+ years of age with arthritis and overweight/obesity who reported receiving provider weight-loss counseling [23]. While this notable, the increase in those receiving counseling was much less among adults aged 65 and older (4.2 percentage point increase; from 36.4% to 40.6%) [23].

The percentage of older adults is generally higher in rural areas than in urban areas in the US [24,25], and there is evidence that the prevalence of obesity and chronic diseases related to obesity, including arthritis [10] are higher in rural areas compared to urban areas [1,24,26,27,28]. Furthermore, there are well-established rural-urban disparities in the availability and quality of health care for older adults [29]. For example, rural hospitals were rated statistically poorer on seven of eight quality indicators than urban hospitals [30]. Availability of primary care is also worse in rural areas compared to urban areas [31]. Boring and colleagues analyzed the 2015 Behavioral Risk Factor Surveillance System (BRFSS) data to determine rural-urban differences in the prevalence of arthritis, with counties being classified into six categories on a rural-urban gradient. They determined that with increasing rurality, the prevalence of arthritis increased, with one in three adults (aged 18+) living in the most rural counties reporting that they had been told by a doctor or other health professional that they had some form of arthritis, gout, lupus, or fibromyalgia [10]. Among adults aged 65+, 54.7% in the most rural areas reported having arthritis compared to 49.7% in the most urban areas (large metropolitan city center) [10]. 

Given the aging US population, rising rates of obesity among older adults with arthritis, and the benefits associated with weight loss for individuals with obesity and arthritis, the objective of this study was to determine if there are differences by rural-urban status among older adults with obesity and arthritis in being advised by a health care provider to lose weight. The study hypothesis was that increasing urbanity would be associated with greater likelihood of receiving a recommendation from a health care provider to lose weight.

## 2. Materials and Methods

This is a secondary analysis of data from the BRFSS, the largest system of health-related telephone surveys administered by the Centers for Disease Control and Prevention (CDC). The BRFSS, a random-digit dialing telephone-based health survey, collects self-reported health data from adults 18+ years old across the US, and is used to identify emerging health problems, track progress on meeting health objectives, and evaluate public health policies and programs. All de-identified data are available on the CDC’s web site.

The data analyzed for this study were collected as part of the 2011 BRFSS, these data were used as this is the most recent year in which the county-level information (which is needed to determine rural-urban status) was available in the BRFSS data set (n = 506,467). Five states (MI, MN, WI, SC, TN) included the BRFSS module that assessed arthritis status in 2011 (n = 5739). The analytic sample was restricted to adults residing in these five states who were between 60–79 years of age (n = 8002) and then to respondents with a body mass index (BMI) ≥ 30 kg/m^2^ who reported having arthritis and had no missing values for the key study variables (final sample n = 2920). Each BRFSS respondent’s county of residence (as recorded in the BRFSS) was linked to their county’s population density (decile) obtained from the US Bureau of the Census.

The 2011 BRFSS uses raking weighting which adjusts for individual demographic variables including type of telephone (landline or mobile) in a series of data processing–intensive iterations. When each variable in the weighting process is entered in the model, the weights are adjusted until the sample weights are representative of the population, which increases the representativeness of and reduces potential bias [32].

### 2.1. Measures

*Weight status and arthritis status:* Self-reported height and weight were used to determine BMI, and the analytic sample was limited to participants with a BMI ≥ 30 kg/m^2^. Arthritis status was assessed by a single item that asked: “Have you ever been told by a doctor or other health professional that you have some form of arthritis, rheumatoid arthritis, gout, lupus, or fibromyalgia?” with the response options yes or no.

*Receipt of weight loss recommendation (WLR):* Respondents who reported receiving a diagnosis of arthritis were asked to respond to the following question: “Has a doctor or other health professional ever suggested losing weight to help your arthritis or joint symptoms?” (yes, no). Individuals who answered “yes” were classified as having received a weight loss recommendation (WLR).

*Rural-urban status:* Population density is often used to assess rural-urban status in the public health literature [28], and this measure was obtained by linking the respondent’s county of residence to the corresponding county-level population density from the 2010 US Decennial Census. For this analysis, population density for the counties in the states that administered the BRFSS module was divided into quintiles, with quintile 5 being the most urban. Population density could have been divided differently (e.g. tertiles, quartiles), but quintiles were selected for ease of interpretation while allowing detecting potentially non-linear differences in rural-urban status. 

*Demographics:* Examined demographics included gender, age, race/ethnicity (white/non-Hispanic, black/non-Hispanic, Hispanic, “other”); marital status (married, divorced, widowed, separated, never married, partnered); education (high school or less, high school graduate, attended college/technical school, graduated college/technical school); and income (<$15,000, ≥15,000 to <$25,000, ≥$25,000 to <$35,000, ≥$35,000 to <$50,000, and ≥$50,000).

### 2.2. Analyses

Descriptive statistics were obtained for all key exposure variables, including frequencies for categorical variable (receipt of WLR, race/ethnicity, marital status, education, and income), standard deviations (age, BMI) or interquartile range (rural-urban status) for continuous variables. A generalized linear model (GLM) was constructed to examine the association between receipt of a WLR and population density controlling for demographics. The model was limited to respondents with complete data for all examined study variables. In addition, the model used population density quintile as an indicator variable for population density quintile to account for potential non-linear associations between rural-urban status and odds of a receipt of a WLR. All analyses were conducted using SPSS version 24.0 (IBM Corp., Armonk, NY, USA) and controlled for the complex survey design. Statistical significance was set to *p* < 0.05. 

## 3. Results

As seen in Table 1, the sample was 83.6% white, 57.8% female, and 20.0% had less than a high school diploma. Of all respondents, 63.2% reported that they had received a provider WLR for their arthritis. More than 60% of individuals living in the most urban areas (quintile 4: 62.6%, quintile 5: 66.1%) reported receiving a WLR compared to 55.6%–58.1% of those in the more rural areas (quintiles 1–3). In addition to rural-urban differences, there were differences in receipt of a provider WLR by demographic characteristics. More women (66.0%) than men (59.3%) reported receiving a WLR and more respondents aged 60–64 received a WLR (67.4%) than older respondents (range 59.0% to 67.4%). Black respondents (70.7%) were more likely to receive a WLR than whites (62.8%), Hispanics (34.4%), and those identifying as multicultural (67.4%) or “Other” (61.9%). There also were differences by education: 51.2% of respondents with less than high school diploma reported receiving a WLR compared to 63.1% of high school graduates, 70.1% of respondents with some college and 65.4% of college graduates.

As seen in Table 2, results of the adjusted GLM model determined that receipt of WLR increased with increasing urbanicity (*p* for trend <0.01). That is, respondents in quintiles 2–5 were more likely than those living in quintile 1 (most rural) to have reported receiving a WLR. The results of the GLM models determined that adults aged 60–64 and women were most likely to have received a WLR. Analysis also determined that increased educational attainment (more than a high school diploma) was associated with an increased likelihood of receiving a WLR.

## 4. Discussion

The current study identified rural-urban differences in receipt of a WLR for older adults who have arthritis and obesity, with respondents living in more urban counties being more likely to have received a WLR from a healthcare provider. This finding suggests education and outreach efforts designed to increase the percentage of healthcare providers who recommend weight loss to patients with obesity and arthritis may need to differ by location with additional emphasis on providers practicing in more rural areas. Recent analysis of 2015 BRFSS data found that ≥50% of adults aged 65+ reported having arthritis, and that the prevalence rate was greatest in the most rural counties [10]. 

The analytic sample was limited to individuals with obesity, yet only 63.2% of respondents reported receiving a WLR. The percentage concurs with or is higher than previous research. For example, a recent study found that less than 50% of adults aged 65+ with arthritis received weight loss counseling, but this sample include individuals with overweight and obesity [21]. Given that obesity among older adults contributes to age-related declines in health [5], this is concerning. There is a clear need to increase the number of older adults with obesity who receive provider counseling, especially given increasing rates of obesity among older adults with arthritis [9].

Healthcare providers may be hesitant to suggest weight loss to older adults with arthritis due to bone mineral density and osteoporosis concerns. Nonetheless, intentional moderate weight loss can be beneficial for arthritis symptoms and is not associated with significant changes in bone health if weight bearing exercises also are recommended and followed [33,34]. When recommending weight loss to older adults, providers need to be cognizant of the fact that weight loss may promote loss of muscle mass [35] and sarcopenia [36]. Therefore, providers should recommend increasing physical activity, including participation in weight bearing exercises, in addition to providing weight loss counseling. Some health care providers may be reluctant to recommend that their older patients lose weight due to the “obesity paradox,” which suggests that older adults with obesity are more likely to develop cardiovascular disease but are less likely to die from this disease [37]. However, Bowmen et al. (2017) found that after controlling for confounders, obesity among older adults was associated with a reduced lifespan and increased coronary heart disease and type 2 diabetes [38]. Moreover, providers may be reluctant to address weight loss due to unrecognized ageism or the belief that being a healthy weight is less important once over 65 years of age. It also is possible that providers in more rural areas may be reluctant to make recommendations for weight loss due to the perceived lack of community-based resources. Future research could explore these issues.

In addition to rural-urban and age differences in the receipt of a provider WLR, there were notable differences by education. Individuals with lower levels of educational attainment (≤high school diploma) were less likely to receive a WLR than were respondents who had attended some or graduated from college. Other studies, not limited to older adults, also have found that individuals with higher levels of education are more likely to receive weight loss advice than those with lower levels of education [18,20], although they are less likely to have arthritis [10]. Furthermore, study results indicate differences in receipt of provider WLR by race/ethnicity. Black respondents were more likely to report receipt of a provider WLR than white respondents, as were multiracial respondents and those classified as “Other”, although all respondents had obesity. This finding concurs with other studies [18], and it has been hypothesized the providers are more cognizant of obesity risks among racial/ethnic minorities. However, importantly, Hispanic respondents in this study were less likely than whites to receive a WLR, which contrasts with other studies [18]. It should be noted that the sample was primarily white (83.6%) and only 10.9% Black, 1.4% “Other”, and 2.0% Hispanic. In addition, women were more likely to have been advised to lose weight than men. 

Time constraints, competing demands, provider’s discomfort addressing weight status, and limited nutrition education [39] may have contributed to the absence of a WLR. Increasing provider self-efficacy to address weight loss and behavior change during and after medical training (e.g., medical school, physician assistant, nursing, and nurse practitioner programs) could contribute to increased confidence and ability in addressing obesity with patients and making needed recommendations [39,40]. In addition to recommending weight loss, providers also need to be able to provide patients with evidence-based strategies for behavior change. For example, patients could be given evidence-based informational handouts that explain the benefits of self-monitoring while suggesting weight loss strategies, including the use of apps to track behaviors such as physical activity and diet as well as weight. This type of advice could be offered by trained medical assistants. In addition, environmental changes such as the use of automated reminders in electronic medical records could contribute to an increase in needed WLRs. The use of interdisciplinary medical teams that include dietitians and exercise physiologists for more seamless referrals and ease of care of the whole person also should be encouraged [41]. 

Study findings should be considered in light of study limitations, which include the cross-sectional study design and the use of self-reported data to determine weight status, arthritis status, and receipt of a WLR. It is possible that the reliance on self-reported measures may over or underestimate the obesity and arthritis status, both of which were used to determine the analytic sample: however, the item used to assess arthritis has been determined to be reliable and adequate for surveillance purposes [42].

In addition, the current study was limited to the five states that include the examined BRFSS items in 2011. Furthermore, an additional study limitation is the BRFSS response, which may impact the validity of the findings. The median state response rate for mobile and landline phones was 73.8%. 

Nonetheless, recent research indicates that counseling for weight loss by healthcare providers remains low [17]. It is, however, possible that providers suggested increasing physical activity or making dietary changes over recommending weight loss. Study strengths include a large sample size and use of a large, nationally representative data set. In addition, this is the first study, to our knowledge, to look at differences in rural-urban status in recommendations for weight for older adults with obesity and arthritis.

## 5. Conclusions

In summary, despite known benefits of weight loss, receipt of a WLR from healthcare providers for individuals with arthritis declined with age, although the sample was limited to respondents with obesity. Notable rural-urban differences were identified: those in more urban counties were more likely to have a provider’s WLR. Receipt of a WLR declined with age and individuals with less education and men were less likely to receive a WLR. Reasons for these differences should be explored in future research and to understand why only about two-thirds (63.2%) of respondents with arthritis and obesity were advised to lose weight to ameliorate their arthritis and associated symptoms.

## Figures and Tables

**Table 1:** Prevalence of receipt of weight loss recommendations (WLRs) by study characteristics among adults with arthritis and obesity, Behavioral Risk Factor Surveillance System, 2011.

Characteristics		N = 2920	Weighted %	Receipt of WLR (Weighted %)
**Age**	60–64	998	36.0	67.4
	65–69	863	29.0	59.4
	70–74	646	19.7	64.2
	75–79	413	15.2	59.0
**Sex**	Male	984	42.2	59.3
	Female	1936	57.8	66.0
**Education**	<High school (HS)	398	20.0	51.2
	HS grad	1081	34.7	63.1
	Some college	810	29.9	70.1
	College grad	625	15.2	65.4
	Missing	6	0.2	54.8
**Race/ethnicity**	White	2226	83.6	62.8
	Black	509	10.9	70.7
	Other	57	1.4	61.9
	Multiracial	55	1.0	67.4
	Hispanic	29	2.0	34.4
	Missing	39	0.9	67.2
**Income**	<$15,000	415	9.7	68.1
	$15,000–<25,000	686	24.1	61.1
	$25,000–<35,000	401	15.6	67.8
	$35,000–<50,000	416	14.0	67.1
	$50,000+	593	21.1	59.6
	Missing	409	15.6	59.8
**Population density quintile**	1 (rural)	10	0.2	58.1
	2	236	6.1	56.9
	3	476	14.0	55.6
	4	637	22.4	62.6
	5 (urban)	1424	57.2	66.1

Note: Percentages may not add up to exactly 100 per cent, owing to rounding off.

**Table 2:** Adjusted and weighted odds ratios of receipt of weight loss recommendation (WLR) among adults with arthritis and obesity, Behavioral Risk Factor Surveillance System, 2011.

Characteristics		N = 2920	Odds Ratio (95% Confidence Interval) *
**Age group**	60–64	998	1 (referent group)
	65–69	863	0.757 (0.749, 0.766)
	70–74	646	0.883 (0.871, 0.894)
	75–79	413	0.726 (0.716, 0.737)
**Sex**	Male	984	1 (referent group)
	Female	1936	1.297 (1.285, 1.309)
**Education**	<High school (HS)	398	1 (referent group)
	HS grad	1081	1.536 (1.516, 1.556)
	Some college	810	2.198 (2.167, 2.228)
	College grad	625	1.991 (1.958, 2.025)
**Race/ethnicity**	White	2226	1 (referent group)
	Black	509	1.287 (1.267, 1.306)
	Other	57	1.311 (1.256, 1.369)
	Multiracial	55	1.026 (0.979, 1.074)
	Hispanic	29	0.369 (0.357, 0.381)
**Income**	<$15,000	415	1 (referent group)
	$15,000–<25,000	686	0.696 (0.684, 0.708)
	$25,000–<35,000	401	0.930 (0.912, 0.948)
	$35,000–<50,000	416	0.705 (0.691, 0.719)
	$50,000+	593	0.528 (0.518, 0.538)
	Missing	409	0.595 (0.584, 0.607)
**Population density quintile**	1 (rural)		1 (referent group)
	2	10	1.215 (1.105, 1.336)
	3	236	1.148 (1.045, 1.262)
	4	476	1.424 (1.297, 1.565)
	5 (urban)	637	1.668 (1.519, 1.832)
	*p*-value for trend		<0.001

Notes: All analyses were weighted and accounted for the complex study design. * Generalized linear models (GLM) were constructed to examine the association between receipt of a WLR and population density controlling for demographics.
